# Cigarette Smoke-Exposed *Candida albicans* Increased Chitin Production and Modulated Human Fibroblast Cell Responses

**DOI:** 10.1155/2014/963156

**Published:** 2014-09-11

**Authors:** Humidah Alanazi, Abdelhabib Semlali, Laura Perraud, Witold Chmielewski, Andrew Zakrzewski, Mahmoud Rouabhia

**Affiliations:** ^1^Groupe de Recherche en Écologie Buccale, Faculté de Médecine Dentaire, Université Laval, 2420 rue de la Terrasse, Québec, QC, Canada G1V 0A6; ^2^Genome Research Chair, Department of Biochemistry, College of Science, King Saud University, Riyadh 12371, Saudi Arabia

## Abstract

The predisposition of cigarette smokers for development of respiratory and oral bacterial infections is well documented. Cigarette smoke can also contribute to yeast infection. The aim of this study was to investigate the effect of cigarette smoke condensate (CSC) on *C. albicans* transition, chitin content, and response to environmental stress and to examine the interaction between CSC-pretreated *C. albicans* and normal human gingival fibroblasts. Following exposure to CSC, *C. albicans* transition from blastospore to hyphal form increased. CSC-pretreated yeast cells became significantly (*P* < 0.01) sensitive to oxidation but significantly (*P* < 0.01) resistant to both osmotic and heat stress. CSC-pretreated *C. albicans* expressed high levels of chitin, with 2- to 8-fold recorded under hyphal conditions. CSC-pretreated *C. albicans* adhered better to the gingival fibroblasts, proliferated almost three times more and adapted into hyphae, while the gingival fibroblasts recorded a significantly (*P* < 0.01) slow growth rate but a significantly higher level of IL-1*β* when in contact with CSC-pretreated *C. albicans*. CSC was thus able to modulate both *C. albicans* transition through the cell wall chitin content and the interaction between *C. albicans* and normal human gingival fibroblasts. These findings may be relevant to fungal infections in the oral cavity in smokers.

## 1. Introduction

Mucosal candidiasis, especially the oropharyngeal type (OPC), is a common opportunistic infection in both immunocompromised and immunocompetent persons [1]. The leading cause of candidiasis is* Candida albicans*, a commensal dimorphic yeast that colonizes up to 60% of normally healthy individuals [2]. Symptomatic OPC appears under a number of predisposing conditions [3, 4]. From these, tobacco smoking was considered predisposing to oral candidiasis [5]. Indeed, epidemiological studies in immunocompromised patients have identified tobacco as a major risk factor for symptomatic infection [6, 7]. Furthermore, the rate of oral candidal carriage in tobacco smokers was reported to be higher in smokers than in nonsmokers [8, 9]. This may explain why 98% of Indian villager's smokers suffer candidal leukoplakia that can be resolved after cessation of tobacco [10, 11].

The exact mechanism by which candidal carriage may be affected by tobacco is still to be discovered. Previous studies have demonstrated that smoking leads to innate immune reduction facilitating candida colonization and host infection [12]. This may suggest the use of tobacco compounds by* C. albicans* as nutritional factors, since aromatic hydrocarbons contained in cigarette smoke can be converted by* Candida* species to carcinogen end products [13]. It has also been reported that* C. albicans* can catalyze the formation of* N*-nitrosobenzylmethylamine supporting the high candidal leukoplakia level in smokers [14].

During the development of candidiasis,* C. albicans* adheres to and invades the tissue [15, 16]. Tis adhesion is promoted by yeast cell wall proteins [17].* Candida* cell walls contain glucans, mannans, glycoproteins, and chitins [18–20]. In* C. albicans*, three genes encoding different chitin synthases (*CHS1*,* 2*, and* 3*) were identified. Chitin production is dependent on the experimental culture condition. Indeed, the* CHS2* and* CHS3* are preferentially expressed under hyphal culture condition [21, 22], whereas* CHS1* remained at low levels in both yeast and hyphae [23]. Interestingly, Chs1p was found to be essential for cell integrity and virulence [23]. Mutants defective in chitin proteins are less virulent than the parental strain in a mouse model. Thus, with high level of chitin,* C. albicans* can overcome cigarette smoke effects and escape the host immune defense [22].

Gingival fibroblasts are major actor in the host immune defense against* Candida* infection [24, 25]. Gingival fibroblasts play role in tissue structure and function [26]. They are active in the inflammatory response by secreting inflammatory cytokines, such as IL-6 and IL-8, as well as IL-1*β* in response to stimuli that include periodontopathic bacteria [27, 28]. The secretion of these cytokines may, thus, be modulated when fibroblasts are exposed to cigarette smoke.

Exposure to smoke-derived toxicants has been shown to lead to immune dysfunction [29]. Smokers thus run a greater risk of contracting invasive diseases caused by various bacterial pathogens [30–32]. Exposure to cigarette smoke has also been shown to induce the formation of biofilm by various oral/respiratory pathogens* in vitro*, including* P. gingivalis*,* S. aureus*,* S. pneumoniae*,* Klebsiella pneumonia*, and* P. aeruginosa*, as well as* Streptococcus mutans* [30–32]. Cigarette smoke was also reported to increase* C. albicans* adhesion and growth, as well as biofilm formation [33, 34]. These effects were supported by the overexpression of* EAP1*,* HWP1*, and* Sap2* genes known to be active players in* C. albicans* virulence [34]. By modulating* C. albicans* adhesion, proliferation, and biofilm formation* in vitro*, cigarette smoke may also modulate the yeast interaction with human cells such as fibroblasts. The aim of this study was thus to investigate the effect of cigarette smoke condensate on* C. albicans* transition from blastospore to hyphal form, its response to environmental stress, its production of cell wall chitin, and its interaction with human gingival fibroblasts.

## 2. Experimental Protocol

### 2.1. *Candida albicans*

*C. albicans* SC5314, known to be strongly invasive [35], was cultured for 24 h on Sabouraud dextrose agar plates (Becton Dickinson, Oakville, ON, Canada) at 30°C. For the* C. albicans* suspensions, one colony was used to inoculate 10 mL of Sabouraud liquid medium supplemented with 0.1% glucose, pH 5.6. The cultures were then grown in a shaking water bath for 18 h at 30°C, after which time the yeast cells were collected, washed with phosphate-buffered saline (PBS), counted by means of a haemocytometer, and adjusted to 10^6^/mL prior to use.

### 2.2. Gingival Fibroblast Isolation and Culture

Small biopsies of lamina propria tissue (gingival connective tissue) were collected from healthy nonsmokers (18–25 years old) following their informed consent in accordance with Laval University Ethics Committee guidelines. To isolate the gingival fibroblasts, the connective tissue was placed in a collagenase P solution (0.125 U/mL; Boehringer Mannheim, Laval, QC, Canada) for 45 min at 37°C under gentle agitation. The isolated cells (2 × 10^5^) were seeded in 75-cm^2^ flasks (Falcon, Becton-Dickinson, Cockeysville, MD, USA) and grown in Dulbecco's modified Eagle's (DME) medium (Invitrogen) containing 10% fetal calf serum (Invitrogen Canada Inc., Burlington, ON, Canada). Upon 90% confluence, the gingival fibroblasts were used between passage two and four for the different experiments.

### 2.3. Preparation of the Cigarette Smoke Condensate

1R3F cigarettes were purchased from the Kentucky Tobacco Research & Development Center (Orlando, FL, USA) and used to prepare the cigarette smoke condensate solution. Each cigarette was placed into one end of a silicone tube linked to an Erlenmeyer flask containing 200 mL of 0.09% sodium chloride. On the other end, a second silicone tube linked to the Erlenmeyer was connected to a standard vacuum. The cigarette was attached to the cigarette holder and lit and the smoke was extracted by applying vacuum, pulling the smoke directly into the 0.09% sodium chloride solution. The procedure was repeated with a total of ten whole cigarettes. The resulting cigarette smoke condensate (CSC) solution was then sterilized by filtration through a 0.22-*μ*m filter and subsequently stored at 4°C until use.

### 2.4. Effect of CSC on* C. albicans* Transition from Blastospore to Hyphal Form

To determine the effect of CSC on the yeast-to-hyphae transition we used qualitative and quantitative assays.* C. albicans *(10^5^ cells) was grown in 3 mL of Sabouraud dextrose broth supplemented with 0.1% glucose and 10% fetal bovine serum (FBS) with or without CSC at various concentrations (10, 30, and 50%). The negative controls refer to the* C. albicans* cultures without CSC. Different conditions were tested: (1) the hyphae-inducing conditions were previously reported [30], consisting of culture medium supplemented with 10% fetal calf serum followed by incubation at 37°C; (2) culture at 37°C, without serum; (3) culture in the presence of 10% serum at 30°C; and (4) culture at 30°C without serum. Following incubation for 3 or 6 h, the cultures were observed microscopically and photographed to record* C. albicans* morphology (*n* = 5). The density of the* C. albicans* transition was also measured. Furthermore, the length of the hyphal forms in each condition was measured by means of NIH-ImageJ software.

### 2.5. Effect of CSC on* C. albicans* Response to Stressful Agents

To investigate the effect of CSC on* C. albicans* sensitivity/resistivity, we exposed the yeast cells to oxidative, osmotic, or heat stress. To do so,* C. albicans* cells were cultured in Sabouraud with or without CSC at various concentrations (10, 30, or 50%). The culture medium was adjusted to have the same level of nutriments as did the control (absence of CSC). Cultures were incubated in a water bath at 30°C for 24 h under agitation. Following the incubation, the cells were counted with a haemocytometer, after which time 10^6^ cells/mL were treated with hydrogen peroxide (5, 10 or 50 mM) or NaCl (1.2 M) or heat treated at 45°C. Incubation under oxidative, osmotic, or heat stress took place for 60 min under agitation. The cells were then washed twice with sterile PBS and suspended thereafter in Sabouraud medium at 10^6^ cells/mL. The sample volume referring to 10^3^ or 10^4^ of each condition was spotted on Sabouraud agar and incubated at 30°C and colony growth was monitored at 24 and 48 h. Culture on a PDA plate lacking the disrupting agents was also performed and used as a growth control. Cell viability following contact with oxidative, osmotic, or heat stress was evaluated by means of the (3-(4,5-dimethylthiazol-2-yl)-2,5-diphenyl tetrazolium bromide) (MTT) assay (Sigma-Aldrich, St. Louis, MO, USA) which measures viable cells as a function of mitochondrial activity as we previously reported [34]. Absorbance (optical density, OD) was subsequently measured at 550 nm by means of an xMark microplate spectrophotometer (Bio-Rad, Mississauga, ON, Canada).

### 2.6. Effect of CSC on* C. albicans* Cell Wall Chitin Content

#### 2.6.1. Cell Wall Isolation and Purification

*C. albicans* in Sabouraud medium was cultured overnight in the presence or absence of CSC at various concentrations (0, 10, 30, or 50%). Sabouraud medium was supplemented or not with 10% fetal calf serum for hyphae culture conditions. Cells were collected by centrifugation at 1200 rpm for 10 min and were then used to extract cell wall proteins. The* C. albicans* pellets were suspended in 500 *μ*L of sterile PSB and supplemented with 200 *μ*L of glass beads (0.425–0.6 mm in diameter; Sigma-Aldrich, G9268). The samples were cooled at −80°C for 60 sec before being subjected to disruption by means of a MiniBead-beater (Biospec Products, Bartlesville, OK, USA) for 2 min at 5,000 rpm. The samples were then cooled for 2 min at −80°C and the disruption cycle was repeated 15 times. The cells were then washed twice with 1 M NaCl and extracted thereafter in an SDS-MerOH extraction buffer (50 mM Tris, 2% sodium dodecyl sulphate (SDS), 0.3 M *β*-mercaptoethanol, and 1 mM EDTA; pH 8.0) at 100°C for 15 min. The resulting pellets were washed three times with 1 mm of PMSF in distilled water and the walls were freeze-dried, weighed, and used to determine the chitin level in each condition.

#### 2.6.2. Chitin Quantification

The amount of chitin present in the cell wall of each sample was determined as described previously [36]. Briefly, 50 mg of each cell wall were hydrolyzed in 6N HCl for 16 h at 100°C. Following evaporation at 65°C, the samples were dissolved in sterile water (1 mL). From this solution, 0.1 mL was supplemented with 0.1 mL of 1.5N Na_2_CO_3_ in 4% acetylacetone and boiled (100°C) for 30 min, followed by the addition of 0.7 mL of 96% of ethanol to each sample. A volume of 0.1 mL of a solution containing 1.6 g of dimethylaminobenzaldehyde in 30 mL of HCl and 30 mL ethanol was added to each sample and the mixture was incubated for 1 h at 37°C in a dark atmosphere. Samples (3 × 200 mL) were aliquated into a 96-well plate at 200 *μ*L/well and three wells per condition. Absorbance was measured at 520 nm by means of an xMark microplate spectrophotometer (Bio-Rad, Mississauga, ON, Canada) and compared with a standard curve of 0–200 mg of glucosamine. The glucosamine level in each sample was presented as the percentage of cell wall dried weight (*n* = 4).

### 2.7. Evaluation of CSC-Pretreated* C. albicans* Adherence to a Human Gingival Fibroblast Monolayer

To determine whether CSC promoted* C. albicans* adherence to gingival fibroblasts, cells were seeded into 6-well plates and grown at 37°C in a 5% CO_2_ atmosphere until confluence, which was usually reached after four to five days.* C. albicans* was first treated with CSC at various concentrations for 24 h then washed twice with CSC-free culture medium. From this, 10^5^ yeast cells were put in contact with a gingival fibroblast monolayer for 3 and 6 h at 30°C. Following each culture period, the infected and noninfected fibroblast monolayers were washed three times with PBS and subjected thereafter to crystal violet staining. One milliliter of 1% w/v crystal violet solution in demineralized water was then added and the cultures were further incubated at room temperature for 15 min. Following incubation, the nonbound dye was removed from the wells by thorough washing with demineralized water, followed by drying at 37°C. Stained cells were then observed under an inverted microscope and the adherent* C. albicans* cells/clusters were counted and plotted as the number per mm^2^ of fibroblast monolayer culture.

### 2.8. Effect of CSC on the Interaction of* C. albicans* with Gingival Fibroblasts

#### 2.8.1. Evaluation of CSC-Pretreated* C. albicans* Transition following Contact with Human Gingival Fibroblasts

To determine whether the CSC pretreatment enhanced* C. albicans* transition when cultured with human gingival fibroblasts,* C. albicans* was grown in the presence of CSC at various concentrations for 24 h; 10^5^ CSC-pretreated yeast cells were layered onto a confluent fibroblast monolayer under hyphae-promoting conditions. This refers to the presence of 10% serum in the medium and incubation at 37°C [37] for 6 h. At the end of this incubation period, the cultures were washed twice with warm PBS and subsequently stained with crystal violet dye, as described above. The stained cells were then observed and photographed (*n* = 4).

#### 2.8.2. Evaluation of CSC-Pretreated* C. albicans* and Gingival Fibroblasts Growth

To determine the effect of CSC-pretreated* C. albicans* on gingival fibroblast growth and* vice versa*, a transwell (contact-independent) culture system was used. The lower chamber of this culture system was seeded with gingival fibroblasts. When the cultures reached confluence, CSC-pretreated* C. albicans* was seeded inside the upper chamber and integrated to the culture system. The interaction of the gingival fibroblasts with the* C. albicans* thus took place through the culture medium. The cultures were maintained at 37°C in a 5% CO_2_ humid atmosphere for either 24 or 48 h. Following each culture period, the* C. albicans* in the upper chamber was collected separately, and cell density was determined by MTT staining, as described above. As for the gingival fibroblasts, they were collected from the lower chamber and their growth was determined using a haemocytometer (*n* = 5).

### 2.9. Effect of CSC-Pretreated* C. albicans* on IL-1*β* Secretion by Gingival Fibroblasts

To evaluate the level of IL-1*β* secreted by gingival fibroblasts stimulated or not with CSC-pretreated* C. albicans*, supernatants were collected from each condition after 24 and 48 h and were analyzed by sandwich enzyme-linked immunosorbent assay (ELISA, R&D Systems, Minneapolis, MN, USA). The supernatants were collected in tubes containing one *μ*L of a protease inhibitor cocktail for specific use with mammalian cell and tissue extracts (Sigma-Aldrich). Immediately after, the supernatants were centrifuged and used to measure IL-1*β* levels. ELISA plates were read at 450 nm and analyzed by means of a Microplate Reader Model 680 (Bio-Rad, USA). The minimum detectable concentration was under 1 pg/mL, as reported by the manufacturer. Each experiment was repeated four times and the means ± SD were calculated and presented.

### 2.10. Statistical Analyses

Each experiment was performed at least four times, with experimental values expressed as means ± SD. The statistical significance of the differences between the control (absence of CSC, and or absence of* C. albicans*) and the test (presence of CSC alone or CSC-pretreated* C. albicans*) values was determined by performing a one-way ANOVA. Posteriori comparisons were performed using Tukey's method. Normality and variance assumptions were verified using the Shapiro-Wilk test and the Brown and Forsythe test, respectively. All of the assumptions were fulfilled. *P* values were declared significant at ≤0.05. The data were analyzed by means of the SAS version 8.2 statistical package (SAS Institute Inc., Cary, NC, USA).

## 3. Results

### 3.1. Cigarette Smoke Condensate Promoted* C. albicans* Transition from Blastospore to Hyphae Forms

**Figure 1:**
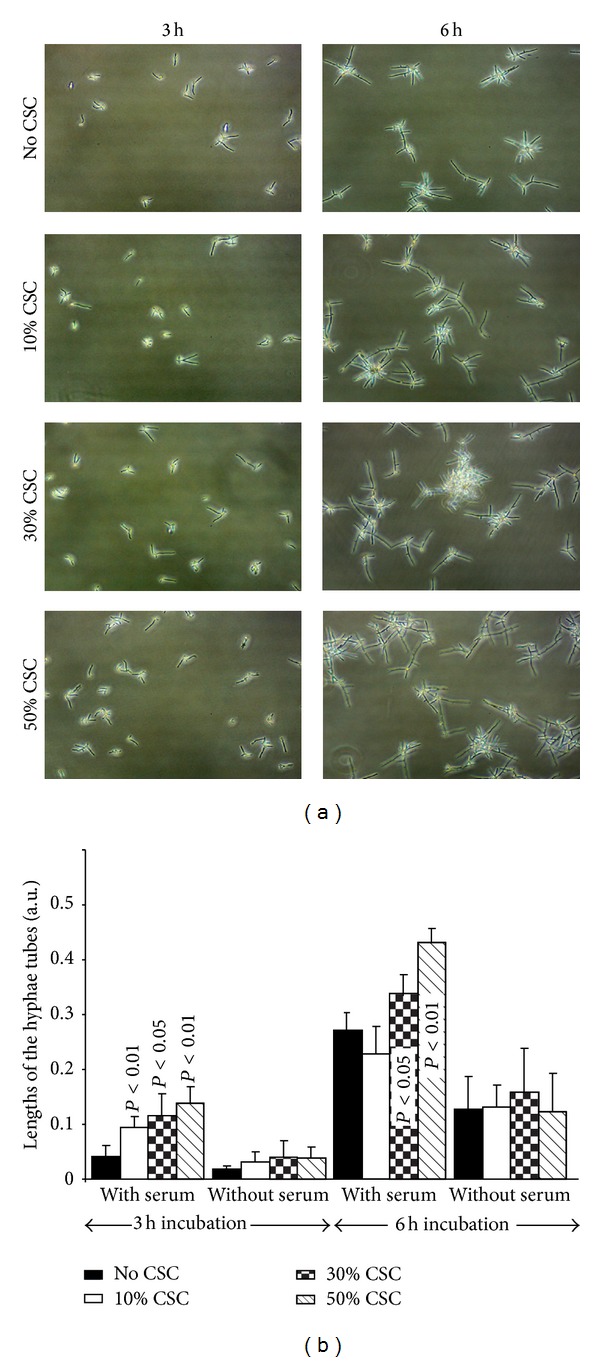
*C. albicans* transition from blastospore to hyphal form following contact with CSC.* C. albicans* was cultured with or without CSC at various concentrations for 3 or 6 h at 37°C in a culture medium supplemented with 10% serum. Following each time point, the cultures were observed under an inverted microscope and photographed (panel (a)). Hyphal tube length was measured by means of NIH-ImageJ software (*n* = 5) and presented (panel (b)). Significance was obtained by comparing the CSC-pretreated and untreated* C. albicans*.

Previously we reported that CSC promoted* C. albicans* growth [34].* Candida* growth was supported by the transition from blastospore to hyphal form. As shown in Figure 1(a), form change was basically observable at 6 h of culture under specific hyphal conditions (presence of serum at 37°C). Indeed, hyphae numbers appeared to be greater with CSC than with the control. The transition was obtained with all CSC concentrations with the incubation at 37°C. Incubation time was also a factor, as hyphal forms were well-defined and were more frequent at 6 h than at 3 h of incubation. Hyphae size also differed in the presence of CSC when* C. albicans* was cultured in the presence of serum at 37°C. As shown in Figure 1(b), longer hyphal tubes were found at 3 and 6 h. It is important to note that CSC induced longer hyphae at as early as 3 h of incubation, with a significantly (*P* < 0.05) longer size observed compared to the control (absence of CSC). The hyphal tubes were longer at 6 h of incubation than at 3 h. Overall data thus demonstrate that cigarette smoke favored* C. albicans* growth and transition.

### 3.2. CSC Decreased* C. albicans* Resistance to Oxidative Stress and Increased Its Resistance to Osmotic and Heat Stress

**Figure 2:**
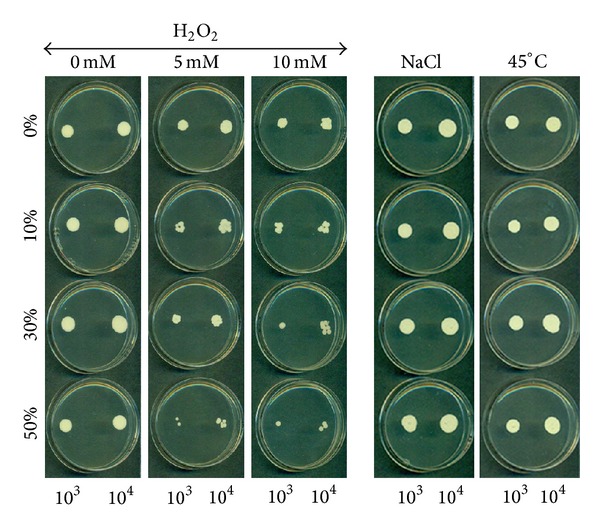
Susceptibility of CSC-treated* C. albicans* to a cell surface-disrupting oxidative, osmotic, and thermal stress agents. Cells were treated or not with CSC then washed and suspended in H_2_O_2_, NaCl, or heat (45°C) for 60 min and then washed and spotted onto Sabouraud agar. Note the increased sensitivity of the CSC-treated* C. albicans* to 5 and 10 mM of H_2_O_2_, but the increased resistance was to osmotic and thermal stresses.

**Figure 3:**
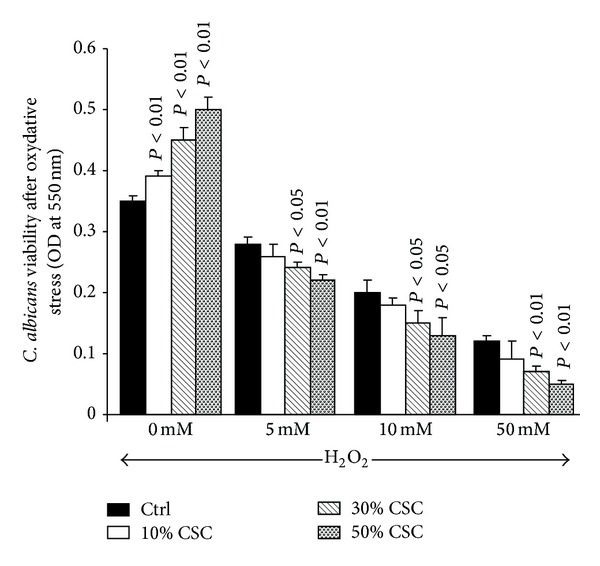
Oxidative stress reduced the growth of the CSC-pretreated* C. albicans*. CSC-pretreated* C. albicans* was washed and 10^6^ cells/mL were incubated with various concentrations of H_2_O_2_ for 1 h. The cells were then washed twice with warm medium and used to ascertain cell viability by MTT assay (*n* = 5). Significance was determined by comparing the CSC-pretreated and untreated* C. albicans*.

**Figure 4:**
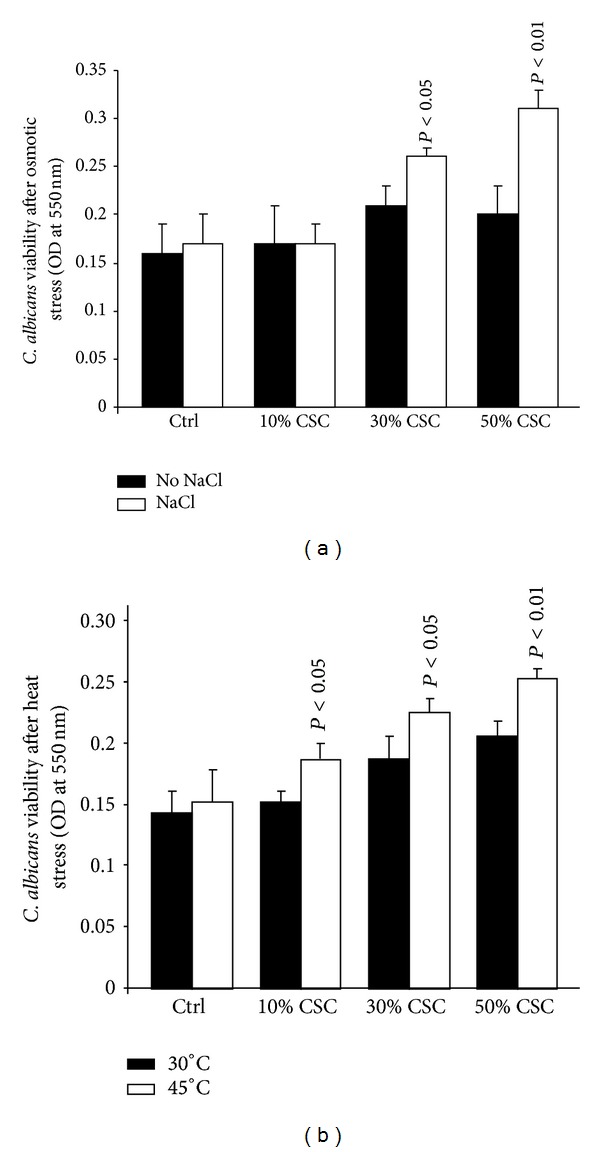
Osmotic and heat stresses increased the growth of CSC-pretreated* C. albicans*. CSC-pretreated* C. albicans* was washed and 10^6^ cells/mL were incubated with NaCl (1.2 M) or at 45°C for 1 h. The cells were then washed twice with warm medium and used to ascertain cell viability by MTT assay (*n* = 5). Significance was determined by comparing the CSC-pretreated and untreated* C. albicans*.

As shown in Figure 2, following one hour of contact with 5 or 10 mM of hydrogen peroxide,* C. albicans* showed a marked decrease in resistance. Without CSC, a reduced colony size was observed following treatment, compared to nontreated samples. The decrease in colony size was greater when* C. albicans* was precultured for 24 h with CSC. The effect was dependent on the hydrogen peroxide concentrations. It should be noted that at 50 mM of hydrogen peroxide, no colony was observed with either 5 or 10 *μ*L of seeding volume (data not shown). Interestingly, when CSC-treated* C. albicans* was subjected to osmotic stress, an increased resistance was observed. As shown in Figure 2, colony size was greater with 1.2 M NaCl treated* Candida* as compared to nontreated cells. The same observation can be made with heat stress (Figure 2). These data were supported by cell viability as ascertained by MTT assay. As shown in Figure 3, hydrogen peroxide significantly (*P* < 0.05) decreased the cell viability of nonprimed or CSC-primed* C. albicans*. However, the decrease in cell viability was greater following hydrogen peroxide stress. Furthermore, this reduced cell viability was dependent on the hydrogen peroxide concentration used (Figure 3). Contrary to oxidative stress, osmotic and heat stress had no negative effect on cell viability. As shown in Figure 4(a), osmotic stress led to a significant (*P* < 0.05) increase of* C. albicans* density, particularly with high levels of CSC (30 and 50%). The same effect was obtained under heat stress (Figure 4(b)).

### 3.3. Exposure of* C. albicans* to CSC Increased Chitin Content

**Figure 5:**
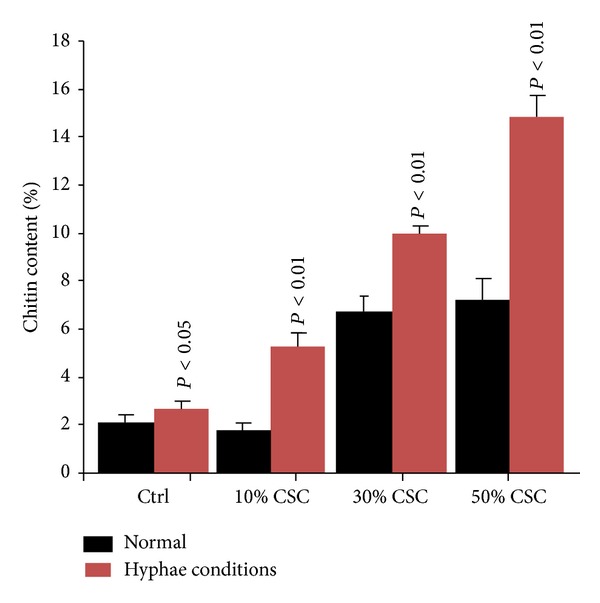
Chitin content in the nonexposed and CSC-exposed* C. albicans*. The glucosamine released from the purified cell walls following acid hydrolysis was quantified and expressed as a percentage of cell wall dried weight. Data presented are from four independent assays performed in triplicate. Significance at *P* < 0.05 was determined by comparing the nonexposed and CSC-exposed yeast cells.

Figure 5 reveals that the growth of* C. albicans* yeast cells in the presence of CSC resulted in elevated chitin content. Exposure of* C. albicans* to CSC under a non-hyphae-inducing culture condition (absence of serum and culture at 30°C) showed a slight increase and significant chitin content in CSC-exposed* C. albicans*, compared to that observed with the nonexposed cells. Chitin production thus increased under normal hyphae culture conditions compared to nonhyphae culture conditions, suggesting that hyphal conditions promoted chitin synthesis. When exposed to CSC,* C. albicans* cells showed significantly (*P* < 0.01) greater levels of chitin than did the control. This increase was more important with higher concentrations of CSC (Figure 5).

### 3.4. CSC-Pretreated* C. albicans* Adhered Better and Changed Form When in Contact with the Gingival Fibroblast Monolayer

**Figure 6:**
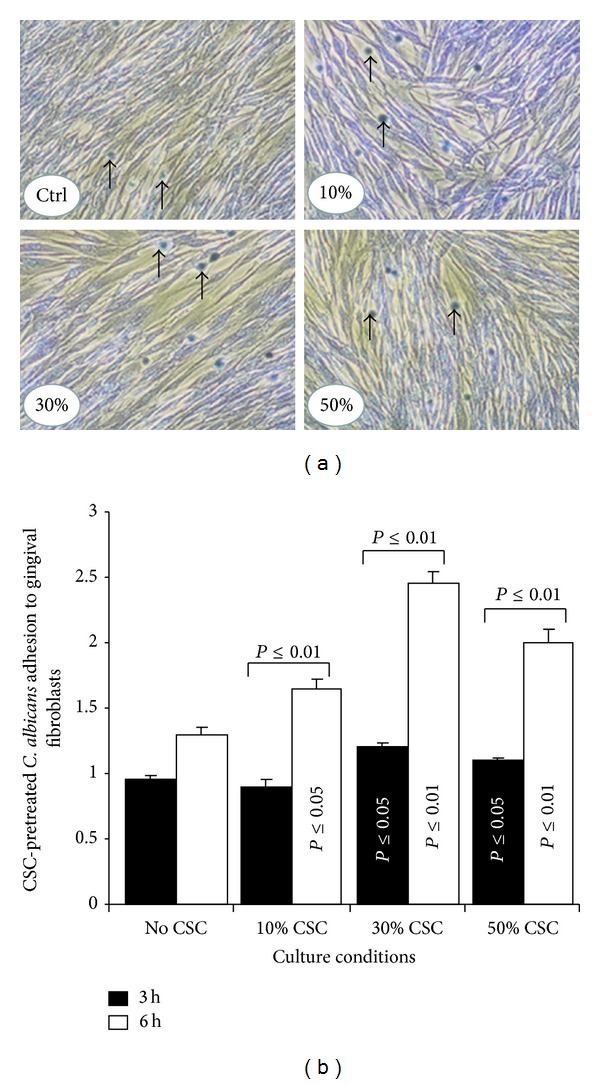
CSC-pretreated* C. albicans* adhered more to gingival fibroblasts.* C. albicans* was incubated for 24 h in the presence of CSC and then used to infect gingival fibroblast cultures for 3 or 6 h. Each culture condition was photographed following crystal violet staining (panel (a), photos after 6 h). Adherent* C. albicans* colonies were counted under an inverted microscope and plotted (panel (b), *n* = 4). Significance was obtained by comparing the CSC-pretreated and untreated* C. albicans*.

**Figure 7:**
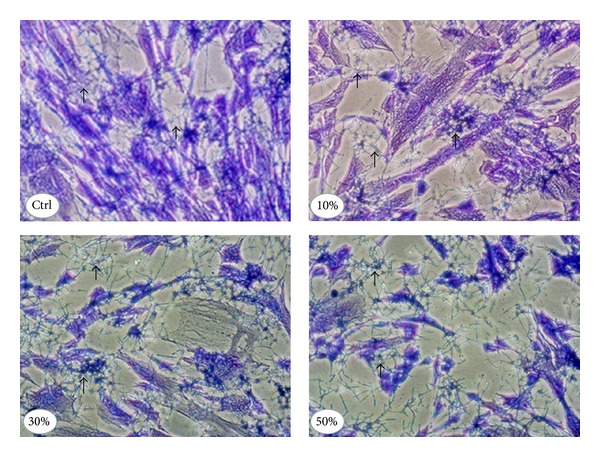
Transition of CSC-pretreated* C. albicans* following contact with gingival fibroblasts.* C. albicans* was incubated for 24 h in the presence of CSC and then used to infect gingival fibroblast cultures for 6 h under hyphal transformation conditions (presence of 10% serum at 37°C). Each culture condition was photographed following crystal violet staining. Note the high level of* C. albicans* hyphae. Representative photos are from 4 different independent experiments.

To investigate whether or not CSC modulated the interaction of* C. albicans* with human gingival fibroblasts,* C. albicans* was pretreated with CSC and its adhesion to a gingival fibroblast monolayer culture at 30°C was analyzed. As shown in Figure 6(a), an elevated number of adherent* C. albicans* colonies were observed in the gingival fibroblast monolayers placed in contact with CSC-pretreated* C. albicans*. These colonies contained more than four yeast cells with a mixture of blastospore and hyphal forms. Quantitative measurement of the colonies per mm^2^ of fibroblast monolayer culture revealed (Figure 6(b)) a significantly high number of colonies in the CSC-pretreated conditions, compared to the CSC-untreated conditions. These high levels of adherent* C. albicans* on the gingival fibroblast monolayers were apparent with each tested CSC concentration. Furthermore, under the hyphae culture conditions, all of the* C. albicans* showed an elongated tube adhering to each culture surface, with a higher hyphal form density observed in the CSC-culture conditions compared to the non-CSC ones (Figure 7). Overall data suggest that CSC promoted* C. albicans* adhesion and transition when interacting with human gingival fibroblast monolayers.

### 3.5. CSC-Pretreated* C. albicans* Proliferated Better When Interacting with the Gingival Fibroblasts

**Figure 8:**
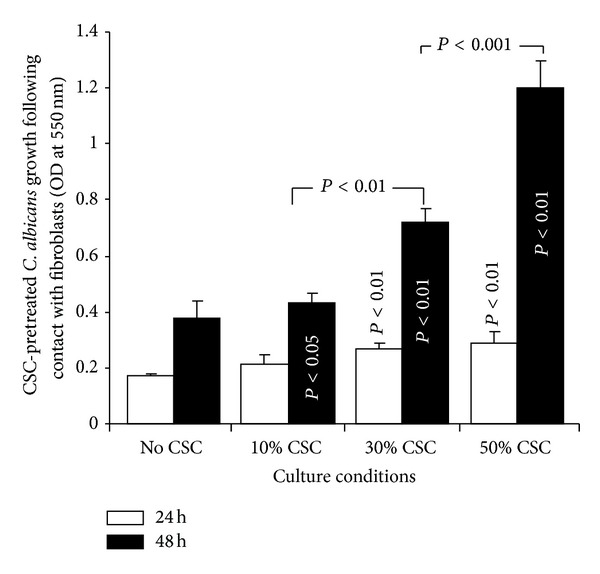
Growth of CSC-pretreated* C. albicans* following contact with gingival fibroblasts.* C. albicans* was incubated for 24 h in the presence of CSC and then used to infect gingival fibroblast cultures for 24 or 48 h at 37°C. The cultures were performed in a transwell culture system allowing for indirect contact between* C. albicans* and the fibroblasts.* C. albicans* cells were then collected and subjected to an MTT colorimetric assay (*n* = 5).

As shown in Figure 8, the proliferation of CSC-pretreated* C. albicans* increased significantly after 24 h of interaction with the gingival fibroblasts. However, this growth was greater at 48 h than at 24 h. Figure 8 shows that a significant (*P* < 0.05) growth increase of* C. albicans* occurred with as low as 10% CSC, compared to the control, at 48 h. The highest growth rates were obtained with 30 and 50% CSC.

### 3.6. CSC-Pretreated* C. albicans* Decreased Gingival Fibroblast Growth and Increased IL-1*β* Secretion

**Figure 9:**
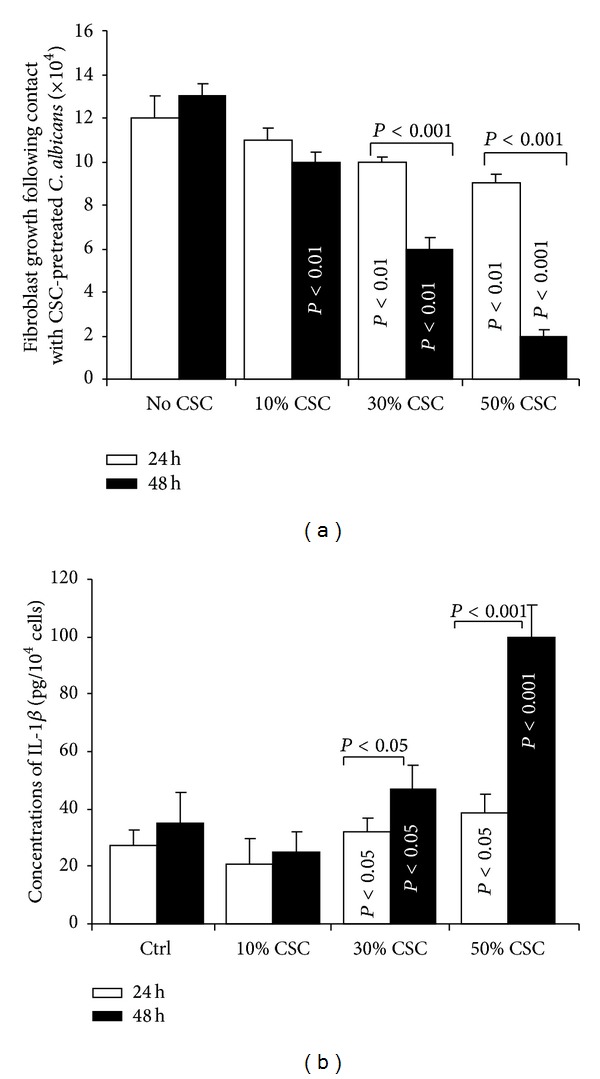
CSC-pretreated* C. albicans* decreased gingival fibroblast growth. Cell cultures were pulsed with CSC-pretreated or untreated* C. albicans* for 24 or 48 h in a transwell culture system (panel (a)) = Fibroblasts were then trypsinized and viable/cell numbers were determined by trypan blue exclusion assay (*n* = 4) (panel (b)) = Culture medium in each condition was used to measure the level of IL-1*β* by ELISA (*n* = 4).

The indirect effect of CSC-pretreated* C. albicans* on gingival human fibroblasts was investigated using a transwell culture system. As shown in Figure 9(a), the number of viable fibroblasts at 24 h was significantly (*P* < 0.01) low with the 30 and 50% CSC concentrations, compared to the control. However, at 48 h, even the 10% CSC concentration led to a significant (*P* < 0.01) decrease in cell numbers. Of interest is that no detaching cells were observed in the cultures with and without CSC-pretreated* C. albicans*. The reduced number was, therefore, not the consequence of apoptotic/necrotic detaching cells but rather the result of a reduction in growth rate. This decreased fibroblast number (due to the interaction with CSC-pretreated* C. albicans*) may lead to the reduction of anti-inflammatory activity of gingival fibroblasts. To test this, we measured the level of IL-1*β* secreted by fibroblasts interacting with CSC-pretreated* C. albicans*. As shown in Figure 9(b), after 24 h of contact, the approximate concentration of IL-1*β* secreted by the gingival fibroblasts infected with CSC-untreated* C. albicans* was 30 pg/10^4^ fibroblasts. This increased to almost 40 pg/10^4^ fibroblasts following contact with 30% CSC-pretreated* C. albicans* and to over 40 pg/10^4^ fibroblasts with 50% CSC-pretreated* C. albicans*. IL-1*β* secretion was greater after 48 h of contact between the fibroblasts and the CSC-pretreated* C. albicans*, reaching close to 100 pg/10^4^ fibroblasts with 50% CSC-pretreated* C. albicans*. The number of viable cells decreased, however, in the presence of CSC-pretreated* C. albicans* (Figure 9(a)) and their secretion of IL-1*β* was elevated (Figure 9(b)).

## 4. Discussion

In a previous study, we demonstrated that the growth rate of* C. albicans* increased when it was cultured in the presence of CSC [34]. This is supported by the present study showing greater* C. albicans* morphological changes in the presence of CSC than in nonexposed controls.

One of the reasons for* Candida* pathogenesis is its ability to interchange between blastospore and hyphal form. Hyphae are considered necessary for invasive growth, while blastospores are considered a clonal expansion form [38]. By promoting* C. albicans* growth and transition, CSC may contribute to* C. albicans* resistance against different stress agents such as oxidative, osmotic, and heat stress. In this study, we demonstrated that CSC-pretreated* C. albicans* was sensitive to H_2_O_2_ but was resistant to osmotic and thermal stress agents. This suggests that treatment with CSC selectively confers resistance of* C. albicans* to some stressful agents but not to others.

It has been reported that* C. albicans* is relatively resistant to reactive oxygen species (ROS), tolerating up to 20 mM of H_2_O_2_ under specific conditions [39]. This oxidative resistance involves AP-1-like transcription factor Cap1 through the Skn7 response regulator [40]; however, these data were generated under adaptive condition of* C. albicans*, which differs from our CSC-exposure experiment. We thus hypothesize that under our conditions,* C. albicans* sensed several stressful components in the CSC and adapted to them, but when in contact with a new and strong stress agent such as H_2_O_2_, it was no longer capable of preventing the damaging effect of this stress agent. Further mechanistic studies will confirm this and shed light on the signaling molecules and death pathway involved. Of interest is that the CSC-pretreated* C. albicans* displayed resistance to osmotic and heat stress. The mechanisms underlying such resistance are still to be investigated. These may include mitogen-activated protein (MAP) kinase pathway involved in osmoadaptation in other yeasts [41, 42]. Further studies are required to demonstrate the signaling mechanisms involved in CSC-pretreated* C. albicans* responses against stresses.

Cell wall proteins are involved in the sensing of stressful agents [43]. These are known to play a key role in yeast cell pathogenesis [43]. Since we demonstrated that CSC-pretreated* C. albicans* produced more chitin, this can be considered as a protective pathway used by* Candida* to overcome the damaging effect of CSC by modulating the cell walls. Indeed, it has been reported that fungi can overcome damaged cell walls by increasing chitin content to maintain cell wall integrity [44].* In vitro* exposure of* C. albicans or A. fumigates* to echinocandins was shown to lead to a compensatory increase in chitin content [45, 46]. Thus the elevated chitin biosynthesis demonstrated in the present study may be considered as a potential mechanism of resistance or tolerance against the effects of CSC. This may translate to a significant clinical impact for smokers, as* C. albicans* cells with a high level of chitin reportedly reduced susceptibility to antifungi such as caspofungin and echinocandins [47].

Cell wall proteins are known to play key role in the interaction between the yeast cells and the host through adhesion processes [17].* C. albicans* adhesion to host cells/tissue is a key factor promoting oral yeast carriage and possible infections [48]. This adhesion may be increased in the presence of cigarette smoke, as demonstrated in the present study, in which CSC-pretreated* C. albicans* adhered better to gingival fibroblast monolayers than did CSC-untreated* C. albicans*. CSC has been shown to favor the adhesion of* S mutans* to dental materials [49] and of* C albicans* on various materials [49]. We thus demonstrate, for the first time, the significant adhesion of* C. albicans* to gingival fibroblasts following contact with CSC. This supports previously reported research with cigarette smoke chemicals, showing the influence of nicotine on bacterial adhesion to soft tissues [50].

By adhering to gingival fibroblasts,* C. albicans* may have the appropriate conditions to proliferate. Indeed, the present study shows the increased growth of CSC-pretreated* C. albicans* when in contact with human gingival fibroblasts. This may be due to the susceptibility of fibroblasts to CSC-pretreated* C. albicans* as previously reported with non-CSC-pretreated yeast cells [51].

Contact with host cells can be mediated by the fungal cell wall, an essential structure that provides physical strength and protects the fungus from hostile environments [52], including* C. albicans* protection against host cell inflammatory response [53]. The mechanisms underlying this process must be further examined.

The indirect interaction of gingival fibroblasts with CSC-pretreated* C. albicans* led to a decrease in the number of viable gingival fibroblasts. It is suggested that this decrease was not due to cell death through the detachment from the culture plate but was rather, most likely, due to a decrease in the cell growth rate. Further research will undoubtedly shed light on this process.

Interestingly, after being in contact with CSC-pretreated* C. albicans*, viable fibroblasts actively secreted IL-1*β*. Indeed, when comparing Figure 9(a) related to the cell growth and Figure 9(b) related to IL-1*β* secretion, a reverse image can be seen, where the lower number of viable fibroblasts corresponds to a high level of IL-1*β* secretion. The importance of IL-1*β* in protecting the mammalian host from invasive* C. albicans* infection has been clearly demonstrated, as mice deficient in IL-1*β* experienced decreased survival levels and increased fungal burdens relative to wild-type mice [54, 55]. The presence of CSC may thus contribute to the interaction of* C. albicans* with human gingival fibroblasts, leading to fibroblast stimulation and IL-1*β* secretion to overcome the damaging effect of CSC and* C. albicans*, as the* C. albicans* untreated with CSC led to low level of IL-1*β* secretion by the fibroblasts. This high level of IL-1*β* may support fibroblast protection against CSC-pretreated* C. albicans* virulence.

## 5. Conclusion

Cigarette smoke promoted* C. albicans* transition from blastospore to hyphal form. CSC-pretreated* C. albicans* was sensitive to oxidative stress but was resistant to osmotic and heat stresses. The CSC-pretreated* C. albicans* also displayed high levels of chitin, particularly under hyphae culture conditions. In addition, CSC modulated* C. albicans* interaction with the host by increasing the adhesion of this yeast to and proliferation on gingival fibroblasts. Contact with CSC-pretreated* C. albicans* affected the fibroblasts which exhibited a reduced growth rate and increased IL-1*β* secretion. Overall, this study suggests that cigarette smoke may promote* C. albicans* pathogenesis, supporting both the increased persistence of this pathogen in smokers and the severity of candidiasis.
